# [Retracted] circNSUN2 promotes the malignant biological behavior of colorectal cancer cells via the miR‑181a‑5p/ROCK2 axis

**DOI:** 10.3892/or.2023.8672

**Published:** 2023-11-27

**Authors:** Junlin Chi, Shuang Liu, Zhizhong Wu, Yanqiang Shi, Chengmin Shi, Tong Zhang, Binghong Xiong, Yujian Zeng, Xiangqian Dong

Oncol Rep 46: 142, 2021; DOI: 10.3892/or.2021.8093

Following the publication of the above article, a concerned reader drew to the Editor's attention that the data showing the results of TUNEL staining of tumours featured in the four panels of Fig. 2G on p. 4, and potentially some of the photographs of the tumours shown in Fig. 2F, were strikingly similar to data appearing in different form in another article written by different authors that had already been submitted for publication elsewhere prior to the submission of this paper to *Oncology Reports*.

In view of the fact that certain of these data had already apparently been submitted for submission in a different journal, the Editor of *Oncology Reports* has decided that this paper should be retracted from the Journal. After having been in contact with the authors, they accepted the decision to retract the paper. The Editor apologizes to the readership for any inconvenience caused.

